# Strenuous Physical Training, Physical Fitness, Body Composition and Bacteroides to Prevotella Ratio in the Gut of Elderly Athletes

**DOI:** 10.3389/fphys.2021.670989

**Published:** 2021-06-22

**Authors:** Katarína Šoltys, Leonard Lendvorský, Ivan Hric, Eva Baranovičová, Adela Penesová, Ivan Mikula, Miroslav Bohmer, Jaroslav Budiš, Silvia Vávrová, Jozef Grones, Marian Grendar, Martin Kolísek, Viktor Bielik

**Affiliations:** ^1^Department of Microbiology and Virology, Faculty of Natural Sciences, Comenius University in Bratislava, Bratislava, Slovakia; ^2^Comenius University Science Park, Comenius University in Bratislava, Bratislava, Slovakia; ^3^Department of Biological and Medical Science, Faculty of Physical Education and Sport, Comenius University in Bratislava, Bratislava, Slovakia; ^4^Biomedical Center Martin, Jessenius Faculty of Medicine in Matin, Comenius University in Bratislava, Martin, Slovakia; ^5^Institute of Clinical and Translational Research Biomedical Center, Slovak Academy of Sciences, Bratislava, Slovakia; ^6^The Concern Foundation Laboratories at The Lautenberg Center for Immunology and Cancer Research, Israel-Canada Medical Research Institute, Faculty of Medicine, The Hebrew University, Jerusalem, Israel; ^7^Department of Molecular Biology, Faculty of Natural Sciences, Comenius University in Bratislava, Bratislava, Slovakia

**Keywords:** microbiome, exercise, body fat, VO2max, aging

## Abstract

Regular physical activity seems to have a positive effect on the microbiota composition of the elderly, but little is known about the added possible benefits of strenuous endurance training. To gain insight into the physiology of the elderly and to identify biomarkers associated with endurance training, we combined different omics approaches. We aimed to investigate the gut microbiome, plasma composition, body composition, cardiorespiratory fitness, and muscle strength of lifetime elderly endurance athletes (LA) age 63.5 (95% CI 61.4, 65.7), height 177.2 (95% CI 174.4, 180.1) cm, weight 77.8 (95% CI 75.1, 80.5) kg, VO2max 42.4 (95% CI 39.8, 45.0) ml.kg^–1^.min^–1^ (*n* = 13) and healthy controls age 64.9 (95% CI 62.1, 67.7), height 174.9 (95% CI 171.2, 178.6) cm, weight 83.4 (95% CI 77.1, 89.7) kg, VO2max 28.9 (95% CI 23.9, 33.9), ml.kg^–1^.min^–1^ (*n* = 9). Microbiome analysis was performed on collected stool samples further subjected to 16S rRNA gene analysis. NMR-spectroscopic analysis was applied to determine and compare selected blood plasma metabolites mostly linked to energy metabolism. The machine learning (ML) analysis discriminated subjects from the LA and CTRL groups using the joint predictors *Bacteroides* 1.8E + 00 (95% CI 1.1, 2.5)%, 3.8E + 00 (95% CI 2.7, 4.8)% (*p* = 0.002); *Prevotella* 1.3 (95% CI 0.28, 2.4)%, 0.1 (95% CI 0.07, 0.3)% (*p* = 0.02); *Intestinimonas* 1.3E-02 (95% CI 9.3E-03, 1.7E-02)%, 5.9E-03 (95% CI 3.9E-03, 7.9E-03)% (*p* = 0.002), *Subdoligranulum* 7.9E-02 (95% CI 2.5E-02, 1.3E-02)%, 3.2E-02 (95% CI 1.8E-02, 4.6E-02)% (*p* = 0.02); and the ratio of *Bacteroides* to *Prevotella* 133 (95% CI -86.2, 352), 732 (95% CI 385, 1079.3) (*p* = 0.03), leading to an ROC curve with AUC of 0.94. Further, random forest ML analysis identified VO2max, BMI, and the *Bacteroides* to *Prevotella* ratio as appropriate, joint predictors for discriminating between subjects from the LA and CTRL groups. Although lifelong endurance training does not bring any significant benefit regarding overall gut microbiota diversity, strenuous athletic training is associated with higher cardiorespiratory fitness, lower body fat, and some favorable gut microbiota composition, all factors associated with slowing the rate of biological aging.

## Introduction

Systematic and regular physical work and exercise are associated with a plethora of health benefits. They help to sustain or even improve physical and mental functions and moods among the elderly. They can also reverse some chronic disease conditions and help the elderly to stay mobile and self-sufficient (McPhee et al., 2016). Despite the frequently communicated benefits of physical activity and healthy aging, the overwhelming majority of older people in western countries do not meet the globally recommended levels of physical activity necessary to maintain a healthy status (Luzak et al., 2017; UK Chief Medical Officers (CMO), 2019). Insufficient physical activity among middle-aged adults indicates a further need for physical activity promotion in order to gain health benefits. The health-related issues associated with aging involve slow deterioration of the immune system (Sellami et al., 2018), endocrine alterations such as those associated with menopause in women and andropause in men (Janssen, 2016), loss of bone mass or density (osteoporosis) (Hoffman et al., 2019), muscle mass and muscle strength decline (Saini et al., 2009), progressive cognitive decline (Damoiseaux, 2017), and overall deterioration of physical fitness (Shephard, 2009). The most recent findings suggest a link between changes in the composition of gut microbiota and the aging process itself. Thus, the therapeutic potential of microbiome-targeted interventions represents a major focus for research in the field of geriatric and regenerative medicine (Rondanelli et al., 2015; Vaiserman et al., 2017).

Compared to the younger adult population, the elderly show reduced microbiota diversity, characterized by significant interindividual variability, with lower numbers of *Firmicutes*, *Bifidobacteria*, *Clostridium cluster XIV*, *Faecalibacterium prausnitzii*, *Blautia coccoides*, and *Eubacterium rectale* and an increased presence of *Enterobacteriaceae* and *Bacteroidetes* (Rondanelli et al., 2015). In addition to the many publications regarding the benefits of regular physical activity, newly published data show that physical exercise performed at least at the level recommended by the World Health Organization (WHO) can modify the composition of gut microbiota (Bressa et al., 2017). Furthermore, short-chain fatty acids (SCFAs) are the main fermentation products of the gut microbiota and provide the link between the gut microbiota and an individual’s physiology (Nishitsuji et al., 2017). However, there is paucity of information on whether the gut microbiota of elderly athletes engaged in life-long high-endurance exercise training differ from that of seniors whose physical activity meets the minimal requirements recommended by the WHO.

Therefore, the objective of the present study was (a) to perform an observational study comparing the composition of the gut microbiota, selected plasma metabolites, physical fitness, and body characteristics of lifetime high-endurance athletes (LA) lifetime elderly endurance athletes and subjects who meet the minimum recommended physical activity levels (CTRL) (Chodzko-Zajko et al., 2009), and (b) to investigate the association between gut microbiota community structures and body composition, cardiorespiratory fitness, muscle strength, and metabolic characteristics. We hypothesized that the gut microbiota composition of lifetime elderly endurance athletes (LA) and healthy controls (CTRL) would differ. We expected a higher alpha diversity of microbiota in LA defined by the Shannon and Simpson index. We also expected to find specific microbes at different taxonomic levels and metabolites showing associations with metabolic health markers.

## Materials and Methods

### Recruitment and Group Characteristics

#### Cohort Recruitment

The cohort of healthy elderly men was recruited by the staff at the Hamar Institute for Human Performance at the Faculty of Physical Education and Sports of Comenius University in Bratislava, Slovakia during a 2-year period. All of them were Caucasian and inhabitants of Slovakia. In total, 34 subjects were examined, but only 22 met the strict inclusion and exclusion criteria. They were divided into two subgroups: LA (*N* = 13) and CTRL (*N* = 9). All of the participants provided their written informed consent. The study was conducted according to the Helsinki Declaration and approved by the Ethics Committee of the Faculty of Physical Education and Sport Comenius University (FTVS UK-6/19). The inclusion criteria were as follows: (a) all athletes were between 60 and 70 years of age, (b) the LA reported active sport history and structured, lifelong endurance exercise training at least four times per week, and (c) the CTRL reported no active sport history but met the recommendations on physical activity for older adults (Chodzko-Zajko et al., 2009).

The exclusion criteria included previous history of major gastrointestinal surgery, active gastrointestinal bleeding or inflammation; inflammatory bowel disease (IBD); cancer; diabetes mellitus; and without special eating history (e.g., vegan) and poor dietary habits. Subjects were excluded if they had taken antibiotics, antifungal drugs, or probiotics less than 2 months prior to the start of the study; had constipation or diarrhea or some digestive problems less than 3 weeks prior to the start of the study; and healthy elderly subjects who had no active chronic diseases such as hypertension and coronary artery disease, liver disease, infectious diseases, or chronic inflammatory or autoimmune disorders (all diagnosed prior to or less than the 2 months prior to the trial). Smoking, alcohol abuse, or drug abuse were included in the exclusion criteria. Candidates with an inadequate caloric intake and negative energy balance were also excluded.

#### Nutritional Data

Nutritional data were collected by means of a 24-h dietary recording over five consecutive days to assess the participants’ habitual diet. All participants were instructed to measure and monitor complete daily food intake and to make notes after each meal. The recorded information was assessed by a research nutritionist utilizing Planeat software (Planeat s.r.o, Bratislava, Slovakia). As a result, information regarding ingested carbohydrates, proteins, fats, fiber, and energy content was gathered. The food items not listed in the software were manually added as required.

#### Cardiorespiratory and Physical Fitness (VO2max)

To test cardiorespiratory fitness (VO2max), the subjects underwent a maximum incremental test on a bicycle ergometer (COSMED Metabolic Company, Rome, Italy). Environmental conditions were standardized, the temperature was maintained at 20°C, and relative humidity ranged between 50 and 60%. After an initial familiarization, the warm-up was comprised of 5 min of cycling at a power output of 0.5 w.kg^–1^, and the load was increased by 0.25 w.kg^–1^ every min until volitional exhaustion. To record the maximum workload, the last step should have been held for an entire minute. We intended to meet the criteria to achieve maximum effort within 8 to 12 min (American College of Sports Medicine (ACSM), 2013). Confirmation of maximum exhaustion was met if at least one of the two following objective criteria or two of the three following criteria for maximum exhaustion were met: maximum heart rate >200 minus age, RER peak >1.10, and assessment of perceived exertion >18 (Borg Scale 6–20) (Howley et al., 1995; Midgley et al., 2007). Two exercise physiologists supervised the procedures during the testing. VO_2_ data collected during the last 10 s of each workload were averaged.

Knee extension isometric strength was performed in a seated position on a backward-inclined (15°) chair by a dynamometer (S2.0 Science to Practice, Ljubljana, Slovenia). The hips and shoulders were stabilized and secured with safety belts. The rotational axis of the dynamometer was aligned with the transverse knee-joint axis and connected to the distal end of the tibia using a length-adjustable rigid lever arm. The subjects performed a maximum voluntary isometric contraction of the knee extensors twice. The knee-joint angle was 130°. The isometric contractions lasted for 3 s and were separated by a 2-min rest interval. The highest torque (Nm) was recorded as isometric strength performance.

Maximum isometric handgrip force was recorded over 10 s using a handheld hand-grip ergometer (EH101—electronic hand dynamometer, Camry, Hong Kong, China).

#### Body Composition

Anthropometric parameters including weight and height were measured twice and averaged. Body mass index (BMI) was calculated by body weight (in kilograms) divided by height squared (in meters) expressed in units of kg/m^2^. The InBody 720 multifrequency impedance analyzer (Biospace Co., Ltd., Seoul, Korea) was used to measure body fat. InBody testing was performed by using an eight-point tactile electrode method, with the subject standing in an upright position with hands holding the electrodes and feet positioned on the electrodes.

#### Stool and Blood Sample Analysis

Participants were properly instructed on how to avoid contamination during sample collection. They were provided with a DNA/RNA Shield Fecal Collection Tube designed for the collection and preservation of nucleic acids from stool specimens (Zymo Research, Irvine, CA, United States) and a stool collection tube. Samples were stored in DNA/RNA Shield Fecal collection tubes at ambient temperature until delivered to the laboratory. The sample stability at ambient temperature (2 years) guaranteed by the manufacturer was sufficient to ensure sample quality until being processed in the laboratory. Stool collection samples without any preservation buffer were immediately stored at −70°C.

Blood was drawn from all of the study subjects into polyethylene tubes with EDTA as an anticoagulant and immediately processed. After centrifugation at 4°C, all plasma aliquots were stored at −20°C until assayed. Blood and fecal samples were jointly collected from study participants after an overnight fast. Participants were advised to maintain their habitual diet and activity patterns.

#### DNA Extraction, High-Throughput Sequencing, and Bioinformatics

The total DNA from fecal samples was extracted with ZymoBIOMICS DNA/RNA kit (Zymo Research, Irvine CA, United States) according to the manufacturer’s protocol. PCR amplification of 16S rRNA using the 27f-1492r primer set (Lane et al., 1985) was carried out in a reaction mixture containing 1 ng of DNA, 5 × FIREPol Master Mix (Solis BioDyne, Estonia), and 0.2 μM of each primer. The thermal cycling program comprised (1) an initial denaturation step (15 min at 95°C) and (2) 27 cycles of (a) denaturation (20 s at 95°C), (b) annealing (30 s at 60°C), and (c) extension (2 min at 72°C).

After PCR product assessment with agarose electrophoresis, 1 ng of column-purified DNA (DNA Clean and Concentrator-5, Zymo Research) was processed by random fragmentation chemistry Nextera XT DNA library kit (Illumina Inc., San Diego, CA, United States). Low-cycle PCR was used for the indexing of individual samples for further multiplexing. PCR products were purified with Agencourt AMPure XP magnetic beads (Beckman Coulter, Brea, CA, United States). Finalized libraries were quantified fluorometrically using a Qubit 2.0 Fluorometer (Thermo Fisher Scientific, Waltham, MA, United States). DNA profiles of sequencing libraries were verified using an Agilent 2100 Bioanalyzer (Agilent Technologies, Santa Clara, CA, United States) and a High Sensitivity DNA Kit (Agilent Technologies). DNA libraries were analyzed using the Illumina MiSeq platform (Illumina Inc., San Diego, CA, United States) via 300-bp paired-end reads.

#### Blood Plasma Metabolites; NMR Data Acquisition

Plasma fraction was deproteinated by adding 600 μl of methanol to 300 μl of plasma. The mixture was vortexed for a few seconds and stored at −20°C for 20 min. Subsequently, the mixture was centrifuged for 30 min at 14,000 rpm. Finally, 700 μl of supernatant was dried out and mixed with 100 μl of stock solution (150 mM phosphate buffer and 0.3 mM TSP-d_4_ 3-(trimethylsilyl)-propionic-2,2,3,3-d_4_ acid sodium salt as a chemical shift reference in deuterated water) and 500 μl of deuterated water. The final mixture (550 μl) was transferred into a 5-mm NMR tube.

A 600-MHz NMR spectrometer Avance III equipped with cryoprobe (Bruker, Ettlingen, Germany) was used to obtain NMR data. Initial settings were done on an independent sample and adopted for measurements. Before measurement, the samples were stored in Sample Jet at 6°C for not longer than 3 h and randomly ordered for acquisition. Measurements were carried out at 310 K. An exponential noise filter was used to introduce 0.3-Hz line broadening before the Fourier transform. We used standard Bruker profiling protocols with the following modifications: profiling 1D NOESY with presaturation (noesygppr1d): FID size: 64 k, dummy scans: 4, number of scans: 128, spectral width: 20.4750 ppm; COSY with presaturation (cosygpprqf): FID size: 4 k, dummy scans: 8, number of scans: 1, spectral width: 16.0125 ppm; homonuclear *J*-resolved (jresgpprqf): FID size: 8 k, dummy scans: 16, number of scans: 4; profiling CPMG with presaturation (cpmgpr1d, L4 = 126, d20 = 3 ms): FID size: 64 k, dummy scans: 4, number of scans: 128, spectral width: 20.0156 ppm. All experiments were conducted with a relaxation delay of 4 s; all data were zero filled once.

#### Illumina Data Processing

Adapters and low-quality read ends were removed using Trimmomatic (Bolger et al., 2014), based on quality control statistics generated by FastQC (Andrews, 2018). Sufficiently large fragments (>35 bp) from both reads were assembled into contigs with Emirge (Miller et al., 2011). Assembly was guided by known reference sequences from the Silva SSU and LSU database (version 128). Chimeric contigs were further filtered with UCHIME (Quast et al., 2013) to exclude potentially misassembled products. The remaining contigs were annotated with taxonomic labels based on their closest homolog in the Silva database (database sequences were retrieved from EMBL-EBI/ENA and cross-checked with RDP). The observed taxonomy abundances were arranged into hierarchical charts using Krona (Edgar et al., 2011).

#### Statistical and Omics Data Analyses

Statistical analyses were carried out using the SPSS 21.0 program for Windows (SPSS, Inc., Chicago, IL, United States). Data normality was checked through the Shapiro–Wilk test. An independent t-test was conducted to compare parametric data (gut microbiome, body composition, and metabolism). Pearson’s correlation coefficient was used to examine the association between variables (gut microbiome, body composition, and energy metabolism). The significance level of p < 0.05 was applied.

The data were also explored and analyzed in R ver. 4.0.3, Ondov et al. (2011) using libraries beeswarm, randomForestSRC, ggRandomForests, and glmnet [Ehrlinger, 2016; Eklund, 2021; Friedman et al., 2010; R Core Team (RCT), 2020]. Exploratory data analysis involved visualizing the data by swarmplots overlaid with boxplots and summarizing the data by the median and lower and upper quartiles. Data were subjected to two Wilcoxon–Mann–Whitney sample tests and presented as a mean and 95% confidence interval. The elastic network (enet) machine learning (ML) algorithm was used for the identification of potential biomarkers among the metabolomic data. The metabolites identified by enet were then uploaded into the random forest (RF) ML algorithm to obtain the out-of-bag ROC curve and thus an estimate of the discriminative ability of selected metabolites. A preselected subset of the relative abundance of four bacterial genera (*Bacteroides*, *Prevotella*, *Intestinimonas*, and *Subdoligranulum*), the *Bacteroides/Prevotella* ratio, and three fitness parameters (BMI, visceral tissue, and VO2max) were uploaded into an RF ML algorithm in order to assess their predictive performance by the out-of-bag ROC curve and rank them by importance and graph depth. Due to the imbalanced data (13 sportsmen vs 9 controls), an imbalanced version of RF was used.

## Results

### Participants’ Characteristics and Body Composition Analysis

The basic physical characteristics of the 22 subjects in the LA and CTRL groups are summarized in Table 1. Significant (*p* < 0.05) differences were observed in body weight, fat content, and BMI. From the reports, we found the training volume of elderly athletes during an active sports career at younger age 795.4 min/wk (683.2–907.6) and 392.3 min/wk (321.1–463.5) at present.

**TABLE 1:** Physical characteristics of the cohort of analyzed individuals—lifelong endurance athletes (LA, *n* = 13) and control group (CTRL, *n* = 9).

**Variable**	**LA**	**CTRL**	***p-*value**
Age, years	63.5 (61.4–65.7)	64.9 (62.1–67.7)	0.40
Height, cm	177.2 (174.4–180.1)	174.9 (171.2–178.6)	0.27
Weight, kg	77.8 (75.1–80.5)	83.4 (77.1–89.7)	0.05
BMI, kg/m^2^	24.8 (24.0–25.6)	27.3 (24.9–29.7)	0.002
Total body fat,%	19.4 (17.3–21.5)	26.2 (21.9–30.5)	0.002
Visceral body fat, level	9.5 (8.3–10.6)	14.1 (10.6–17.7)	0.003
Muscle mass%	37.44 (34.9–40.0)	34.4 (27.6–44.9)	0.62

*Data are presented as a mean and (95%CI). Differences were considered significant at *p* < 0.05 (adjusted *p*-value corrected for false discovery rate). BMI, body mass index.*

### Dietary Habits

The diets of both groups conformed to the parameters of a balanced diet with an appropriate percentage of macronutrients: 10-15% proteins, 50-60% carbohydrates, and 30-35% lipids. No significantly different macronutrient consumption or total energy intake was detected between the groups.

### Cardiorespiratory Fitness, Isometric Strength, and Handgrip Force

The significant differences in maximum oxygen consumption and mechanical power between the LA and CTRL groups were detected. However, no significant differences in knee extensor strength were detected between the groups; both, left and right handgrip force, were higher in the LA group (Table 2).

**TABLE 2:** Cardiorespiratory fitness, isometric strength, and handgrip force of lifelong endurance athletes (LA, *n* = 13) and control group (CTRLs, *n* = 9).

**Variable**	**LA**	**CTRL**	***p-*value**
VO2max, ml.kg^–1^.min^–1^	42.4 (39.8–45.0)	28.9 (23.9–33.9)	0.0001
PM, w	258.8 (239.5–278.2)	172.8 (131.7–213.8)	0.0001
HRM, bpm	161.1 (148.6–173.6)	157.2 (129.1–185.3)	0.76
IFE, N	397.7 (331.2–464.3)	365.8 (313.6–417.9)	0.52
HGL, N	46.7 (42.6–50.8)	38.9 (32.65–45.1)	0.02
HGR, N	50.1 (45.8–54.4)	41.4 (34.4–48.4)	0.02

*Data are presented as a mean and (95%CI). Differences were considered significant at *p* < 0.05 (adjusted *p*-value corrected for false discovery rate). PM, mechanical power at VO2max; HRM, heart rate at VO2max; IFE, Isometric force of lower legs extensors; HGL left handgrip force; HGR, right handgrip force.*

**FIGURE 1:**
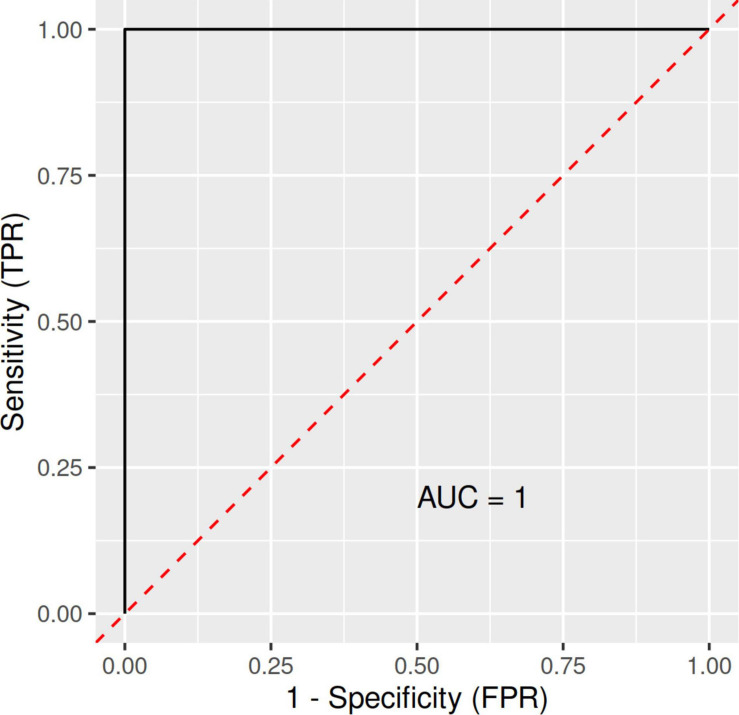
ROC (receiver operating characteristic) curves with an area under the ROC curve (AUC) for the RFM-L algorithm with VO2max, fat content, and BMI as joint predictors/discriminators between LA and CTRL groups. FPR, false-positive rate; LA, lifetime elderly endurance athletes; CTRL, controls; RFM-L, random forest machine-learning; TPR, true positive rate.

A RF and ML analysis with VO2max, fat content, and BMI as joint predictors resulted in an ROC (receiver operating characteristic) curve with an AUC (area under the ROC curve) of 1.00 (Figure 1). Thus, combined, these predictors were a near-perfect discriminator between the CTRL and LA groups.

### Microbiome Composition of Stool Samples

In total, 14 bacterial phyla were detected in the CTRL and LA groups. The dominating phylum [presented as a mean (mean CTRL/mean LA)] in both groups was *Firmicutes* 75% (73.9%/75.6%) followed by *Bacteroidetes* 16.5% (18.6%/14.4%) and *Proteobacteria* 1.0% (0.5%/1.5%). Six phyla including *Actinobacteria* 0.5% (both groups), *Verrucomicrobia* 0.14% (0.06%/0.21%), *Lentisphaerae* 0.008% (0.005%/0.01%), *Synergistetes* 0.008% (0.006%/0.01%), *Cyanobacteri*a 0.002% (both groups), and *Tenericutes* 0.0005% (0.0006%/0.0003%) were present at a level of less than 1% abundance. *Acidobacteria* was detected in 3 of 9 CTRL samples (0.0003%) and in 8 of 13 LA samples (0.0003%). Additionally, *Spirochates* were detected in three samples (1 CTRL/2 LA), *Candidatus Saccharibacteria* in one sample (LA), *Fusobacteria* in one sample (LA), and *Planctomycetes* in one sample (CTRL).

The *Firmicutes:Bacteroidetes* ratio was not altered significantly (*p* = 0.28), since the ratios for the CTRL and LA groups were very similar 4.9 (3.6–6.3) and 7.0 (3.2–10.8) [mean (95% CI)] (Supplementary Figure 1).

Furthermore, detailed analysis at the family level revealed a significantly different abundance of *Bacteroidaceae* in the CTRL group [3.8% (2.7–4.8)] compared to the LA group [1.8% (1.1–2.5), *p* = 0.002] and *Clostridiales Incertae Sedis* XI [CTRL 1.5%E-03 (0.6E-03–2.4E-03), LA 3.6E-03% (2.3E-03–4.7E-03), *p* = 0.01]. An increased amount of *Cytophagia* (*p* = 0.03) in the CTRL group [1.8E-03% (0.2E-04–3.4E-03)] was proved at the class taxonomical level compared to that of the LA group 3.5E-04% (2.5E-05–6.8E-04) Table 3. A trend toward significance was observed also within the *Ruminococcaceae* family (*p* = 0.2) CTRL 6.9% (5.5–8.3), LA 8.0% (6.7–9.4).

**TABLE 3:** Set of microbial taxa (class, order, and family) differentially present in lifelong athletes (LA) and the sedentary elderly (CTRL).

	**LA (%)**	**CTRL (%)**	***p*-value**
*Cytophagia*	3.5E-04 (2.5E-05–6.8E-04)	1.8E-03 (0.2E-03–3.4E-03)	0.03
*Clost_Incertae Sedis XI*	3.6E-03 (2.3E-03–4.7E-03)	1.5E-03 (0.0005–0.0023)	0.01
*Bacteroidaceae*	1.8 (1.1–2.5)	3.8 (2.7–4.8)	0.002

*Data are presented as a mean and (95%CI).*

**FIGURE 2:**
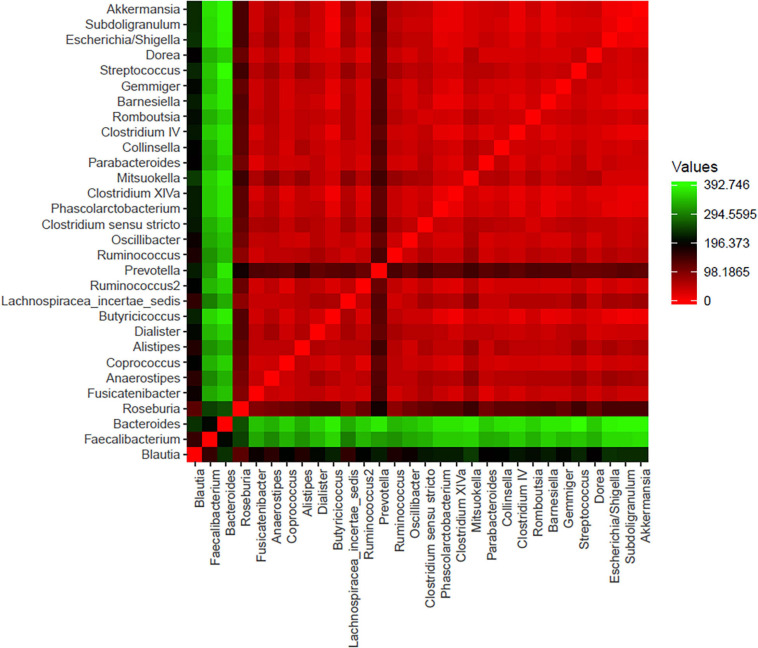
The set of 30 bacterial genera identified as one of the 10 most abundant bacterial genera within at least one sample within both groups of samples. Core microbiome is typical for seniors. The minimum abundance must reach at least 1% of all bacteria and in at least one sample of both groups.

A total of 296 bacterial genera were detected in the analyzed samples: 221 for the CTRL group [111.1 (100.5–121.7)] and 267 for the LA group [on average 121.4 (113.9–128.9)] without significance (*p* = 0.4). The microbial community was dominated by 30 top-most abundant bacteria detected within each sample represented by *Blautia*, *Faecalibacterium*, *Bacteroides*, *Roseburia*, *Fusicatenibacter*, *Anaerostipes*, *Coprococcus*, *Alistipes*, *Butyricicoccus*, *Lachnospiracea incertae sedis*, *Ruminococcus2*, *Prevotella*, *Ruminococcus*, *Oscillibacter*, *Clostridium sensu stricto*, *Clostridium XlVa*, *Parabacteroides*, *Clostridium IV*, *Romboutsia*, *Gemmiger*, *Streptococcus*, *Dorea*, and *Subdoligranulum* that were present in all of the samples of both groups (Figure 2). Except for *Barnesiella* which was not detected in one LA sample, *Dialister*, *Phascolarctobacterium*, *Mitsuokella*, *Collinsella*, *Escherichia/Shigella*, and *Akkermansia* were present within the entire dataset (4 – 18). Significant differences were observed in the relative abundance of genus *Bacteroides* (*p* = 0.002) between the CTRL group [3.8% (2.7–4.8)] and the LA group [1.8% (1.1–2.5), *Phascolarctobacterium* (CTRL 3.2E-02% (1.8E-02–4.6E-02) and (LA 1.6E-01% (0.8E-01–2.4E-01), *p* = 0.1], and *Subdoligranulum* [CTRL 0.03% (0.01–0.04), LA 7.9E-02% (2.5E-02–1.3E-01), *p* = 0.02] Table 4.

**TABLE 4:** Set of microbial taxa (genus) differentially present in lifelong athletes (LA), and the sedentary elderly (CTRL).

	**LA (%)**	**CTRL (%)**	***p*-value**
*Bacteroides*	1.8(1.1−2.5)	3.8(2.7−4.8)	0.002
*Prevotella*	1.3(0.28−2.4)	0.1(0.07−0.3)	0.02
*Intestinimonas*	1.3E-02(9.3E-03−1.7E-02)	5.9E-03(3.9E-03−7.9E-03)	0.002
*Subdoligranulum*	7.9E-02%(2.5E-02−1.3E-01)	3.2E-02%(1.8E-02−4.6E-02)	0.02
*Pseudobutyrivibrio*	3.9E-04(8.7E-05−7.0E-04)	1.3E-03(4.7E-04−2.1E-03)	0.02
*Marvinbryantia*	5.7E-04(2.7E-04−8.7E-04)	1.6E-04(1.1E-05−3.0E-04)	0.02
*Vallitalea*	1.7E-04(2.5E-05−3.1E-04)	0	0.04
*Porphyromonas*	1.4E-04(2.7E-05−2.5E-04)	0	0.04
*Anaerosporobacter*	0	1.2E-04(2.0E-07−2.7E-04)	0.03
*Anaerovorax*	5.0E-03(2.9E-03−6.9E-03)	2.7E-03(1.0E-03−4.4E-03)	0.08

*Data are presented as mean and (95%CI).*

*Blautia* [CTRL 1.6% (1.1–1.9), LA 1.5% (1.1–1.9)], *Faecalibacterium* [CTRL 2.6% (2.2–3.0), LA 2.3% (1.9–2.6)], *Bacteroides*, and *Roseburia* [CTRL 5.2% (3.7–6.6), LA 2.5% (1.7%–3.3%)] formed a significantly different part of all bacteria (*p* = 0.001) 9.3% (7.9–10.8) in the CTRL group compared to 6.2% (5.4–7.2) in the LA group.

The microbiota alpha diversity of elderly athletes defined by the Shannon and Simpson index and the Chao1 index did not differ from that of the controls. Remarkably, a significant (*p* = 0.006) two-fold difference was recorded in the number of singletons in the LA group at the family and order levels, but not at the genus level. Furthermore, deep microbiome investigation revealed several significantly altered bacteria among the control and athlete groups. For the CTRL group, except for *Bacteroides* (mentioned above in the text), also higher abundance (*p* = 0.02) of *Pseudobutyrivibrio* (*Lachnospiraceae*) [CTRL 1.3E-03% (4.7E-04–2.1E-03), LA 3.9E-04% (8.7E-05–7.0E-04)] was detected. *Anaerosporobacter* was found exclusively in the CTRL group [1.2E-04% (2.0E-07–2.7E-04%)]. Four of nine samples were found positive to either *Moryella* (*Lachnospiraceae*) (1/7) or *Anaerosporobacter* (*Lachnospiraceae*) (2/7) or *Mucinivorans* (*Rikenellaceae*) (1/7). Also, the co-occurrence of *Moryella*/*Mucinivorans* (2/7) and *Anaerosporobacter*/*Mucinivorans* (1/7) was observed.

**FIGURE 3:**
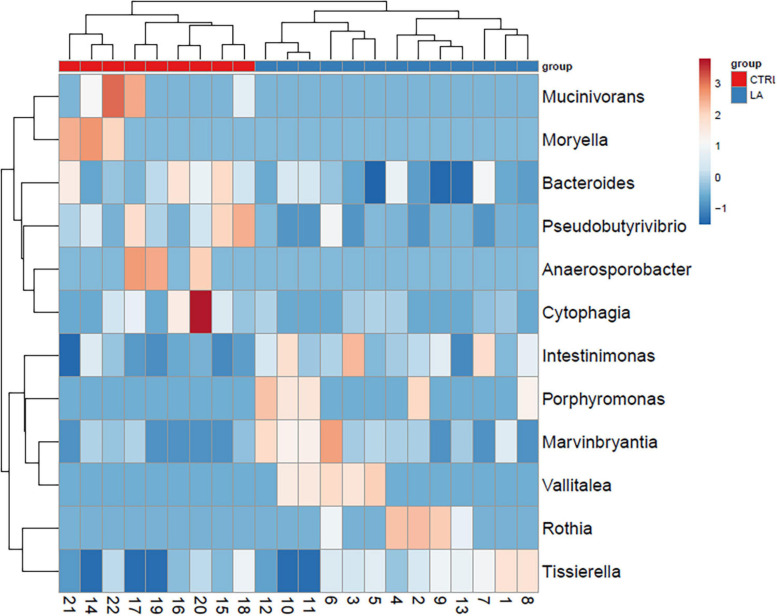
Microbial composition of significantly (*p* < 0.05) distinct bacterial genera within the healthy control group (CTRL) and elderly athletes (LA) seniors. Selected rows are centerd; unit variance scaling is applied to rows. Imputation is used for missing value estimation. Both rows and columns are clustered using correlation distance and average linkage. Numbers represent samples visualized by heat map.

**FIGURE 4:**
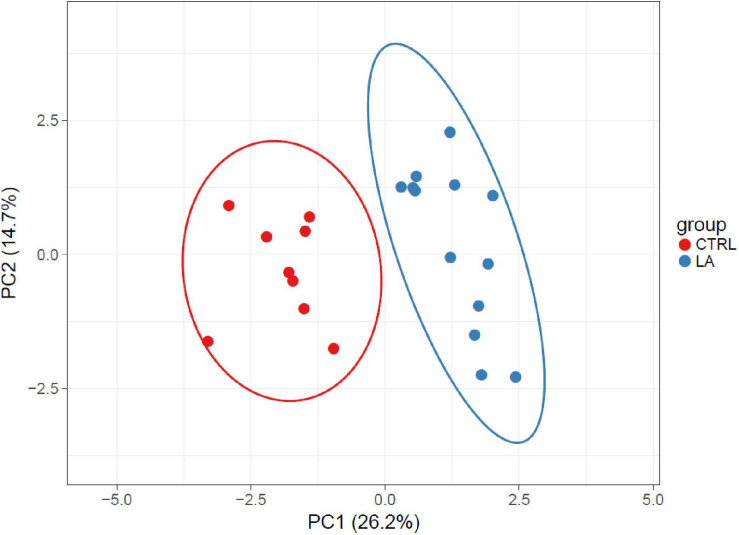
Beta diversity of analyzed samples represented by significantly altered (*p* < 0.05) OTUs in elderly athletes and the control group visualized by PCA. SVD with imputation is used to calculate principal components. X and Y axes show principal component 1 and principal component 2 that explain 26.2 and 14.7% of the total variance, respectively. Prediction ellipses are such that with a probability of 0.95, a new observation from the same group will fall inside the ellipse (*N* = 22 data points).

On the contrary, the microbiome of lifetime elderly athletes was found to possess a higher amount of *Intestinimonas* (5.9E-03% (3.9E-03–7.9E-03), LA 1.3E-02% (9.3E-03–1.7E-02), *p* = 0.002) and *Marvinbryantia* [CTRL 1.6E-04% (1.1E-05–3.0E-04), LA 5.7E-04% (2.7E-04–8.7E-04), *p* = 0.02)] and a trend toward significance considering bacterial genus *Anaerovorax* [2.7E-03% (1.0E-03–4.4E-03), LA 4.9E-03% (2.9E-03–6.9E-03), *p* = 0.08]. Furthermore, the presence of the microbiome *Vallitalea* [LA 1.7E-04% (2.5E-05–3.1E-04)] and *Porphyromonas* [LA 1.4E-04% (2.7E-05–2.5E-04)] was found in the LA group but not in the CTRL group. Selected bacterial genera enabled the discrimination between the CTRL and LA groups (Figures 3, 4).

**FIGURE 5:**
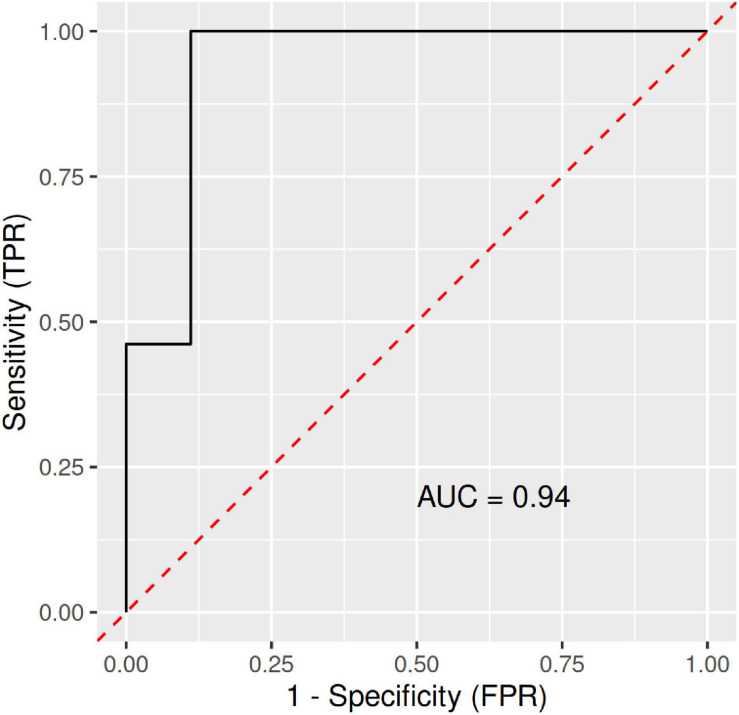
ROC (receiver operating characteristic) curves with an area under the ROC curve (AUC) for the RFM-L algorithm with *Bacteroides*, *Prevotella*, *Intestinimonas*, *Subdoligranulum*, and the *Bacteroides* to *Prevotella* ratio as joint predictors/discriminators between the LA and CTRL groups. FPR, false-positive rate; LA, lifetime elderly endurance athletes; CTRL, controls; RFM-L, random forest machine-learning; TPR, true positive rate.

Furthermore, a significant difference (*p* = 0.03) in the *Bacteroides* to *Prevotella* ratio was observed. While the median *Bacteroides*/*Prevotella* ratio in the CTRL group of 732 (95% CI 385–1079.3) was determined, the same ratio for the LA group was calculated at 133 (95% CI -86.2–352). The ML analysis with *Bacteroides*, *Prevotella*, *Intestinimonas*, *Subdoligranulum*, and the *Bacteroides* to *Prevotella* ratio used as joint predictors led to an ROC curve with an AUC of 0.94, which represents a perfect set of variables to discriminate subjects from the CTRL and LA groups (Figure 5).

**FIGURE 6:**
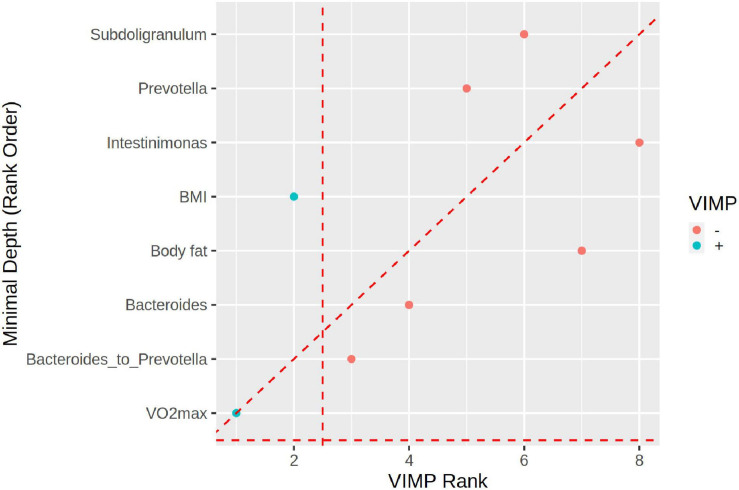
The plot depicting the ranking of predictors by graph depth (*y*-axis) vs. variable importance (VIMP, *x*-axis). VO2max is the best variable for both criteria. The *Bacteroides* to *Prevotella* ratio is the second most important by the VIMP criterion whereas it is the fourth most important by the graph depth. Overall, the two criteria are in good agreement in ranking the predictors.

Additionally, we ranked selected physiological and microbiome markers used in RF ML analyses. While VIMP (RF variable importance) identified VO2max and BMI as the most important markers (the *Bacteroides* to *Prevotella* ratio is third), the GraphDepth analyzer identified VO2max and the *Bacteroides* to *Prevotella* ratio as the most important and BMI as fifth in importance (Figure 6).

### Metabolites

Out of 20 plasma metabolites with an appropriate NMR signal [lactate, alanine, valine, leucine, isoleucine, glucose, acetate, acetone, pyruvate, citrate, phenylalanine, tyrosine, glutamine, lysine, 3-hydroxybutyrate, tryptophane, ketoleucine, ketoisoleucine, ketovaline, and lipoprotein fraction containing (LDL, VLDL, and HDL)], two (acetate and pyruvate) showed statistically significant (*p* < 0.05) and one (lactate) showed near significant (*p* < 0.1) changes between the LA and CTRL groups (all three metabolites were decreased in the LA group in comparison to the CTRL group). The elastic network (ML)-selected metabolites established the LA and CTRL groups with the following importance: acetate (0.65/1) > pyruvate (0.59/1) > alanine (0.5/1) > ketoisoleucine (0.46/1) > lactate (0.32/1) > citrate (0.26/1) > lipoproteins (0.18/1); 1 refers to maximum importance. RF ML analysis performed with the selected metabolites serving as joint predictors revealed low sensitivity, AUC = 0.44; thus, the complex of these metabolites was of no use in predicting whether a senior was from the LA or CTRL group (Supplementary Figure 2).

## Discussion

We performed an observational study to determine the effect of lifetime endurance exercise training on microbiota composition and diversity, cardiorespiratory fitness, and metabolic characteristics in elderly men.

Previous clinical studies reported significant differences in microbial composition associated with aging (Claesson et al., 2011; Xu et al., 2019; Kim and Benayoun, 2020). However, factors other than aging alone might contribute to distinguishing gut microbiota between elderly individuals. Fielding et al. (2019) reported no significant differences of α- and β-diversity when comparing older adults with high and low physical fitness. Similarly, the gut microbiota alpha diversity (defined by the Shannon, Simpson, and Chao1 indexes) of our LA group did not differ from that of the CTRL group. However, changes in the intestinal microbiota of the elderly may not necessarily be affected by aging. They can be consequent to conditions that occur frequently during aging, such as the decline of the general state of health or living conditions, physical activity, and nutrition (Rondanelli et al., 2015). We must underline the fact that the control group in our study was comprised of healthy subjects within the same age range. Furthermore, the CTRL group met the recommended physical activity requirements (Chodzko-Zajko et al., 2009). As expected, we found higher cardiorespiratory fitness (VO2max) in the LA group. However, the cardiorespiratory fitness of the CTRL group was 40% higher than the generally accepted threshold for cardiorespiratory fitness needed to independently perform typical activities of daily living (Arnett et al., 2008). Moreover, we reported no differences in the strength of lower leg extensors between groups. As a result, we concluded that endurance exercise in the elderly performed above global recommendations for physical activity do not bring significant benefits for higher gut microbiota diversity. The microbiome among the elderly displays greater interindividual variation than in younger adults (Claesson et al., 2012). The microbiome composition of older people significantly correlates with measures of frailty, comorbidity, nutritional status, inflammatory markers, and metabolites in fecal water (Claesson et al., 2012). Nevertheless, the impact of changes in microbiota diversity on aging remains poorly understood.

Our deeper microbiome investigation revealed several significantly altered bacteria between the LA and CTRL groups. In accordance with previous studies, we reported a lower relative abundance of genus *Bacteroides* in the LA group. Petersen et al. (2017) found a low abundance of *Bacteroides* in the fecal samples of professional and amateur-level competitive cyclists. Similarly, significantly lower overall abundances of Bacteroides were measured in elite rugby players when compared with low BMI controls (Clarke et al., 2014). At first glance, the results of Yang et al. (2017) are contrary to our results, as they found a positive association between *Bacteroides* and cardiorespiratory fitness in premenopausal women. Moreover, subjects with low aerobic fitness had a lower amount of *Bacteroides*. Although in our study we reported a negative correlation between *Bacteroides* and cardiorespiratory fitness, the variables were presented relative to body weight. However, the results of the abovementioned authors seem to be confounded by adiposity, since all of the differences between premenopausal women disappeared after adjusting for percentage of body fat (Yang et al., 2017). The abundance of *Bacteroides* and their association with physical fitness represents a contradictory issue in literature. Morita et al. (2019) concluded that aerobic exercise training in healthy elderly women may increase intestinal *Bacteroides* in association with improved cardiorespiratory fitness (Morita et al., 2019). In another study, subjects with normal weight and a 2.5-month-long lifestyle intervention showed a decrease in *Bacteroides* by 15.2% with respect to the control group. On the contrary, in the same study, subjects with metabolic syndrome who followed the same lifestyle intervention reported an increase in *Bacteroides* by 1.2%, compared with the control group (Guevara-Cruz et al., 2019). Furthermore, Carbajo-Pescador et al. (2019) emphasized the importance of age in assessing the importance of *Bacteroides*. Based on different results from the aforementioned studies and the results of our study, the abundance of *Bacteroides* to cardiorespiratory fitness could be related to sex and cardiorespiratory fitness level. Male, highly trained young and elderly athletes show lower *Bacteroides* abundance (Clarke et al., 2014; Petersen et al., 2017), but healthy elderly and premenopausal women show higher *Bacteroides* abundance, as a consequence of physical training intervention (Yang et al., 2017; Morita et al., 2019). A significant increase in the relative abundance of *Bacteroides* is also associated with higher consumption of fat in the diet (Murtaza et al., 2019). Several studies indicate a higher prevalence of *Bacteroides* in samples from western countries and industrialized Asian countries, than in samples from less-industrialized and more rural populations (Lin et al., 2013; Nakayama et al., 2015; Obregon-Tito et al., 2015; De Filippis et al., 2016; Shankar et al., 2017). However, in our study we did not report significant differences in the consumed amount of protein and fat between the LA and CTRL groups.

*Prevotella* was another significantly different taxa between the LA and CTRL groups. Using WGS sequencing data, Petersen et al. (2017) split the gut microbiomes of 33 cyclists into three taxonomic clusters, characterized by either high *Prevotella*, high *Bacteroides*, or a mix of many genera including *Bacteroides*, *Prevotella*, *Eubacterium*, *Ruminococcus*, and *Akkermansia*. The high abundance of *Prevotella* was significantly correlated with the amount of time people spent exercising during an average week. A higher abundance of *Prevotella* was also measured in older adults (aged 70–85) who attained better functioning scores measured by a short physical performance battery (Fielding et al., 2019). In any case, genus-level *Prevotella* is not associated only with sport training history. Stroke and transient ischemic attack patients had fewer commensal or beneficial genera including *Prevotella* (Yin et al., 2015), which, along with unclassified *Lachnospiraceae* and unclassified *Ruminococcaceae*, were the main genera contributing to gut composition among non-obese elderly individuals (Zhong et al., 2020). Furthermore, an increased abundance of *Prevotella* was correlated with a number of amino acid and carbohydrate metabolism pathways, including branched-chain amino acid metabolism (Petersen et al., 2017). On the contrary, the abundance of *Prevotella* in faeces correlates with a severe reduction of *Bacteroides* spp. and other genera usually described as beneficial (Arumugam et al., 2011) and is connected to a higher risk for the development of rheumatoid arthritis (Scher et al., 2013). In terms of gut microbiome composition, since *Prevotella* is more dependent on the addition of CO_2_ or bicarbonate (Franke and Deppenmeier, 2018) for biomass formation, long-term favorable conditions must be provided. On the other hand, in general *Prevotella* is associated with healthy fibre-rich diets and with improvement in glucose homeostasis (Kovatcheva-Datchary et al., 2015). *Prevotella* strains are associated with plant-rich diets, but since they are also linked with chronic inflammatory conditions, they are still classified as a potential pathobionts (Kovatcheva-Datchary et al., 2015; Franke and Deppenmeier, 2018).

The evaluation of the *Bacteroides*–*Prevotella* ratio (B/P) as a marker with a predictive potential of weight and fat loss has been carried out in several studies involving humans as well as animals (Lambert et al., 2015; Hjorth et al., 2020). Lower B/P ratios were identified in both normal and diabetic mice that were exposed to regular exercise activity in comparison to sedentary counterparts (Lambert et al., 2015). This is in accordance with the B/P measurements of our study where we reported a lower B/P ratio in the LA group compared to the CTRL group. In another study, the B/P ratio correlated positively and significantly with plasma glucose concentration in a group of 18 subjects diagnosed with *diabetes mellitus* type 2 (Larsen et al., 2010). In addition, B/P counts of another study were negatively correlated with fecal total SCFA in an entire cohort of healthy lean, overweight, and obese participants (Fernandes et al., 2014).

Some short-chain fatty acid-producing bacteria appear to be of higher importance, and their presence is associated with health benefits. We found a higher relative abundance of bacteria characterized by an increased capacity to produce butyrate, such as *Intestinimonas*, in the group of lifetime endurance athletes. Furthermore, in this study *Intestinimonas* was associated with cardiorespiratory fitness when adjusted for weight and presented as an absolute value. *Intestinimonas* is a newly described bacterial genus with representative strains that originate in the mouse and human gut. Despite their remarkable metabolic features including the production of butyrate from both sugars and amino acids, there is only a limited amount of data on their diversity, ecology, and physiology (Bui et al., 2016).

The finding that *Marvinbryantia* was inversely associated with the weight of overweight school-aged children is in accordance with our study where we reported a higher relative abundance of *Marvinbryantia* in the LA group with a lower BMI than the CTRL group (Mbakwa et al., 2018). Further, we found a negative association between *Marvinbryantia* and total body fat. There is no literature describing the association between *Subdoligranulum* and exercise, physical fitness, and body composition. However, a lower score in the total healthy eating index determining diet quality was associated with significantly reduced, relative abundance of *Subdoligranulum* (Liu et al., 2019). Interestingly, *Subdoligranulum* showed a negative correlation with tumor marker alpha-fetoprotein (Zhang et al., 2019). Therefore, we would like to point out that the endurance athletes participating in our study had a higher relative abundance of *Subdoligranulum*, compared to the healthy control group.

The RF and ML analysis identified VO2max, body fat content, BMI, and counts of *Bacteroides*, *Prevotella*, *Intestinimonas*, *Subdoligranulum*, and B/P ratio as suitable, joint predictors with an excellent ability to discriminate between subjects from the LA and CTRL groups. Furthermore, the VIMP and the GraphDepth analysis ranked VO2max, fat content, and the *Bacteroides* to *Prevotella* ratio as the three best-discriminating factors for the subjects from the LA and CTRL groups. As a result, they can be proposed as suitable joint biomarkers discriminating between the physiological status of elderly men for future studies focusing on the physiology and well-being of LA.

We conclude and agree with Barton et al. (2017) that differences in fecal microbiome between elderly athletes and healthy controls show even greater separation at metagenomic levels than at compositional levels and provide additional insight into the diet–exercise–gut microbiota paradigm. Although our findings bring some promising results supporting regular physical activity and sport in elderly subjects, more data is required to better resolve the complex image of microbiome puzzle.

The major limitation of our study is the small number of participants. However, as reported, 34 subjects were examined, but the inclusion and exclusion criteria were very strict. Three men from the control group were diagnosed with prostate cancer, and one from the athlete group was later found on the doping list. In three cases, we were not sure which group to place the individual (e.g., a soldier with a high level of physical fitness who had not played an organized sport). Based on our experience of collecting data over 2 years, it is rare for a subject to be elderly, non-obese, and healthy. Another limitation may be the fact that we did not objectively measure individual domains of sedentary behaviour (sitting at work, watching TV, playing video games, and reading) and other daily physical activities (walking, housework, gardening).

## Conclusion

Our findings suggest that endurance exercise performed the elderly above global recommendations on physical activity do not bring significant benefits for higher gut microbiota diversity.

However, we further suggest that continual exercise by elderly endurance athletes is associated with higher cardiorespiratory fitness, lower body fat, and favorable gut microbiota composition on the lower taxonomy level. The *Bacteroides* to *Prevotella* ratio seems to distinguish the endurance trained elderly from healthy controls. We believe that a stronger, significant difference could be achieved by comparing more heterogeneous groups of elderly subjects.

## Data Availability Statement

The data presented in the study are deposited in the GenBank repository with BioProject accession number PRJNA731908.

## Ethics Statement

The studies involving human participants were reviewed and approved by the Ethics Committee of the Faculty of Physical Education and Sport of Comenius University (FTVS UK-6/19). The patients/participants provided their written informed consent to participate in this study.

## Author Contributions

KŠ writing—review and editing, conceptualization, methodology, project administration, and supervision. LL and IH contributed to experimental design application and design of the work. AP writing—review and editing. EB, MB, and JB acquisition and data analysis and interpretation of data. IM formal analysis, writing—review, and editing. SV and JG acquisition and data analysis and proposal of ideas. MG data analysis, interpretation of data, and preparing figures. MK writing—review and editing and supervision. VB writing—original draft, conceptualization, methodology, project administration, and supervision. All authors contributed to the article and approved the submitted version.

## Conflict of Interest

The authors declare that the research was conducted in the absence of any commercial or financial relationships that could be construed as a potential conflict of interest.

## References

[B1] American College of Sports Medicine (ACSM) (2013). *ACSM’s Guidelines for Exercise Testing and Prescription.* Philadelphia: Lippincott Williams & Wilkins.

[B2] Andrews S. (2018). *FastQC**: A Quality Control Tool for High Throughput Sequence Data.* Available online at: https://github.com/s-andrews/FastQC (accessed January 15, 2021).

[B3] Arnett S. W. Laity J. H. Agrawal S. K. Cress M. E. (2008). Aerobic reserve and physical functional performance in older adults. *Age Ageing* 37 384–389. 10.1093/ageing/afn022 18287178

[B4] Arumugam M. Raes J. Pelletier E. Le Paslier D. Yamada T. Mende D. R. (2011). Enterotypes of the human gut microbiome. *Nature* 473 174–180. 21508958 10.1038/nature09944 PMC3728647

[B5] Barton W. Penney N. C. Cronin O. Garcia-Perez I. Molloy M. G. Holmes E. (2017). The microbiomes of professional athletes differ from those of more sedentary subjects in composition and particularly at the functional metabolic level. *Gut* 67 625–633. 28360096 10.1136/gutjnl-2016-313627

[B6] Bolger A. M. Lohse M. Usadel B. (2014). Trimmomatic: a flexible trimmer for Illumina sequence data. *Bioinformatics* 30 2114–2120. 10.1093/bioinformatics/btu170 24695404 PMC4103590

[B7] Bressa C. Bailén-Andrino M. Pérez-Santiago J. González-Soltero R. Pérez M. Montalvo-Lominchar M. G. (2017). Differences in gut microbiota profile between women with active lifestyle and sedentary women. *PLoS One* 12:e0171352. 10.1371/journal.pone.0171352 28187199 PMC5302835

[B8] Bui T. P. N. Shetty S. A. Lagkouvardos I. Ritari J. Chamlagain B. Douillard F. P. (2016). Comparative genomics and physiology of the butyrate-producing bacterium Intestinimonas butyriciproducens. *Environ. Microbiol. Rep.* 8 1024–1037. 10.1111/1758-2229.12483 27717172

[B9] Carbajo-Pescador S. Porras D. García-Mediavilla M. V. Martínez-Flórez S. Juarez-Fernández M. Cuevas M. J. (2019). Beneficial effects of exercise on gut microbiota functionality and barrier integrity, and gut-liver crosstalk in an in vivo model of early obesity and non-alcoholic fatty liver disease. *Dis. Model. Mech.* 12:dmm039206. 10.1242/dmm.039206 PMC6550047 30971408

[B10] Chodzko-Zajko W. J. Proctor D. N. Fiatarone Singh M. A. Minson C. T. Nigg C. R. Salem G. J. (2009). American College of Sports Medicine position stand. Exercise and physical activity for older adults. *Med. Sci. Sports Exerc.* 41 1510–1530. 19516148 10.1249/MSS.0b013e3181a0c95c

[B11] Claesson M. J. Cusack S. O’Sullivan O. Greene-Diniz R. de Weerd H. Flannery E. (2011). Composition, variability, and temporal stability of the intestinal microbiota of the elderly. *Proc. Natl. Acad. Sci. U. S. A.* 108 4586–4591. 20571116 10.1073/pnas.1000097107 PMC3063589

[B12] Claesson M. J. Jeffery I. B. Conde S. Power S. E. O’Connor E. M. Cusack S. (2012). Gut microbiota composition correlates with diet and health in the elderly. *Nature* 488 178–184. 22797518 10.1038/nature11319

[B13] Clarke S. F. Murphy E. F. O’Sullivan O. Lucey A. J. Humphreys M. Hogan A. (2014). Exercise and associated dietary extremes impact on gut microbial diversity. *Gut* 63 1913–1920. 10.1136/gutjnl-2013-306541 25021423

[B14] Damoiseaux J. S. (2017). Effects of aging on functional and structural brain connectivity. *Neuroimage* 160 32–40. 10.1016/j.neuroimage.2017.01.077 28159687

[B15] De Filippis F. Pellegrini N. Vannini L. Jeffery I. B. La Storia A. Laghi L. (2016). High-level adherence to a Mediterranean diet beneficially impacts the gut microbiota and associated metabolome. *Gut* 65 1812–1821. 10.1136/gutjnl-2015-309957 26416813

[B16] Edgar R. C. Haas B. J. Clemente J. C. Quince C. Knight R. (2011). UCHIME improves sensitivity and speed of chimera detection. *Bioinformatics* 27 2194–2200. 10.1093/bioinformatics/btr381 21700674 PMC3150044

[B17] Ehrlinger J. (2016). *ggRandomForests: Visually Exploring Random Forests.* Available online at: https://cran.r-project.org/package=ggRandomForests (accessed March 10, 2021).

[B18] Eklund A. (2021). *beeswarm**: The Bee Swarm Plot, an Alternative to Stripchart.* Available online at: https://cran.r-project.org/package=beeswarm (accessed March 10, 2021).

[B19] Fernandes J. Su W. Rahat-Rozenbloom S. Wolever T. M. S. Comelli E. M. (2014). Adiposity, gut microbiota and faecal short chain fatty acids are linked in adult humans. *Nutr. Diabetes* 4:e121. 10.1038/nutd.2014.23 24979150 PMC4079931

[B20] Fielding R. A. Reeves A. R. Jasuja R. Liu C. Barrett B. B. Lustgarten M. S. (2019). Muscle strength is increased in mice that are colonized with microbiota from high-functioning older adults. *Exp. Gerontol.* 127:110722. 10.1016/j.exger.2019.110722 31493521 PMC6823114

[B21] Franke T. Deppenmeier U. (2018). Physiology and central carbon metabolism of the gut bacterium Prevotella copri. *Mol. Microbiol.* 109 528–540. 10.1111/mmi.14058 29995973

[B22] Friedman J. H. Hastie T. Tibshirani R. (2010). Regularization Paths for Generalized Linear Models via Coordinate Descent. *J. Stat. Softw.* 33 1–22. 20808728 PMC2929880

[B23] Guevara-Cruz M. Flores-López A. G. Aguilar-López M. Sánchez-Tapia M. Medina-Vera I. Díaz D. (2019). Improvement of Lipoprotein Profile and Metabolic Endotoxemia by a Lifestyle Intervention That Modifies the Gut Microbiota in Subjects With Metabolic Syndrome. *J. Am. Heart Assoc.* 8:e012401. 10.1161/JAHA.119.012401 PMC6755842 31451009

[B24] Hjorth M. F. Christensen L. Kjølbæk L. Larsen L. H. Roager H. M. Kiilerich P. (2020). Pretreatment *Prevotella*-to-*Bacteroides* ratio and markers of glucose metabolism as prognostic markers for dietary weight loss maintenance. *Eur. J. Clin. Nutr.* 74 338–347. 10.1038/s41430-019-0466-1 31285554

[B25] Hoffman C. M. Han J. Calvi L. M. (2019). Impact of aging on bone, marrow and their interactions. *Bone* 119 1–7. 10.1016/j.bone.2018.07.012 30010082

[B26] Howley E. T. Bassett D. R. J. Welch H. G. (1995). Criteria for maximal oxygen uptake: review and commentary. *Med. Sci. Sports Exerc.* 27 1292–1301. 8531628

[B27] Janssen J. A. M. J. L. (2016). Impact of Physical Exercise on Endocrine Aging. *Front. Horm. Res.* 47:68–81. 10.1159/000445158 27348867

[B28] Kim M. Benayoun B. A. (2020). The microbiome: an emerging key player in aging and longevity. *Transl. Med. Aging* 4 103–116. 10.1016/j.tma.2020.07.004 32832742 PMC7437988

[B29] Kovatcheva-Datchary P. Nilsson A. Akrami R. Lee Y. S. De Vadder F. Arora T. (2015). Dietary Fiber-Induced Improvement in Glucose Metabolism Is Associated with Increased Abundance of Prevotella. *Cell Metab.* 22 971–982. 10.1016/j.cmet.2015.10.001 26552345

[B30] Lambert J. E. Myslicki J. P. Bomhof M. R. Belke D. D. Shearer J. Reimer R. A. (2015). Exercise training modifies gut microbiota in normal and diabetic mice. *Physiol. Appl. Nutr. Metab.* 40 749–752. 10.1139/apnm-2014-0452 25962839

[B31] Lane D. J. Pace B. Olsen G. J. Stahl D. A. Sogin M. L. Pace N. R. (1985). Rapid determination of 16S ribosomal RNA sequences for phylogenetic analyses. *Proc. Natl. Acad. Sci. U. S. A.* 82 6955–6959. 10.1073/pnas.82.20.6955 2413450 PMC391288

[B32] Larsen N. Vogensen F. K. van den Berg F. W. J. Nielsen D. S. Andreasen A. S. Pedersen B. K. (2010). Gut microbiota in human adults with type 2 diabetes differs from non-diabetic adults. *PLoS One* 5:e9085. 10.1371/journal.pone.0009085 20140211 PMC2816710

[B33] Lin A. Bik E. M. Costello E. K. Dethlefsen L. Haque R. Relman D. A. (2013). Distinct distal gut microbiome diversity and composition in healthy children from Bangladesh and the United States. *PLoS One* 8:e53838. 10.1371/journal.pone.0053838 23349750 PMC3551965

[B34] Liu Y. Ajami N. J. El-Serag H. B. Hair C. Graham D. Y. White D. L. (2019). Dietary quality and the colonic mucosa-associated gut microbiome in humans. *Am. J. Clin. Nutr.* 110 701–712. 10.1093/ajcn/nqz139 31291462 PMC6736447

[B35] Luzak A. Heier M. Thorand B. Laxy M. Nowak D. Peters A. (2017). Physical activity levels, duration pattern and adherence to WHO recommendations in German adults. *PLoS One* 12:e0172503. 10.1371/journal.pone.0172503 28245253 PMC5330478

[B36] Mbakwa C. A. Hermes G. D. A. Penders J. Savelkoul P. H. M. Thijs C. Dagnelie P. C. (2018). Gut Microbiota and Body Weight in School-Aged Children: the KOALA Birth Cohort Study. *Obesity* 26 1767–1776. 10.1002/oby.22320 30296366 PMC6646907

[B37] McPhee J. S. French D. P. Jackson D. Nazroo J. Pendleton N. Degens H. (2016). Physical activity in older age: perspectives for healthy ageing and frailty. *Biogerontology* 17 567–580. 10.1007/s10522-016-9641-0 26936444 PMC4889622

[B38] Midgley A. W. McNaughton L. R. Polman R. Marchant D. (2007). Criteria for determination of maximal oxygen uptake: a brief critique and recommendations for future research. *Sports Med.* 37 1019–1028. 10.2165/00007256-200737120-00002 18027991

[B39] Miller C. S. Baker B. J. Thomas B. C. Singer S. W. Banfield J. F. (2011). EMIRGE: reconstruction of full-length ribosomal genes from microbial community short read sequencing data. *Genome Biol.* 12:R44. 10.1186/gb-2011-12-5-r44 21595876 PMC3219967

[B40] Morita E. Yokoyama H. Imai D. Takeda R. Ota A. Kawai E. (2019). Aerobic Exercise Training with Brisk Walking Increases Intestinal *Bacteroides* in Healthy Elderly Women. *Nutrients* 11:868. 10.3390/nu11040868 30999699 PMC6520866

[B41] Murtaza N. Burke L. M. Vlahovich N. Charlesson B. O’ Neill H. Ross M. L. (2019). The Effects of Dietary Pattern during Intensified Training on Stool Microbiota of Elite Race Walkers. *Nutrients* 11:261. 10.3390/nu11020261 30682843 PMC6413084

[B42] Nakayama J. Watanabe K. Jiang J. Matsuda K. Chao S.-H. Haryono P. (2015). Diversity in gut bacterial community of school-age children in Asia. *Sci. Rep.* 5:8397. 10.1038/srep08397 PMC4336934 25703686

[B43] Nishitsuji K. Xiao J. Nagatomo R. Umemoto H. Morimoto Y. Akatsu H. (2017). Analysis of the gut microbiome and plasma short-chain fatty acid profiles in a spontaneous mouse model of metabolic syndrome. *Sci. Rep.* 7:15876. 10.1038/s41598-017-16189-5 29158587 PMC5696507

[B44] Obregon-Tito A. J. Tito R. Y. Metcalf J. Sankaranarayanan K. Clemente J. C. Ursell L. K. (2015). Subsistence strategies in traditional societies distinguish gut microbiomes. *Nat. Commun.* 6:6505. 10.1038/ncomms7505 PMC4386023 25807110

[B45] Ondov B. D. Bergman N. H. Phillippy A. M. (2011). Interactive metagenomic visualization in a Web browser. *BMC Bioinformatics* 12:385. 10.1186/1471-2105-12-385 21961884 PMC3190407

[B46] Petersen L. M. Bautista E. J. Nguyen H. Hanson B. M. Chen L. Lek S. H. (2017). Community characteristics of the gut microbiomes of competitive cyclists. *Microbiome* 5:98. 10.1186/s40168-017-0320-4 PMC5553673 28797298

[B47] Quast C. Pruesse E. Yilmaz P. Gerken J. Schweer T. Yarza P. (2013). The SILVA ribosomal RNA gene database project: improved data processing and web-based tools. *Nucleic Acids Res.* 41 D590–D596. 23193283 10.1093/nar/gks1219 PMC3531112

[B48] R Core Team (RCT). (2020). *R: A language and environment for statistical computing.* Vienna: R Foundation for Statistical Computing.

[B49] Rondanelli M. Giacosa A. Faliva M. A. Perna S. Allieri F. Castellazzi A. M. (2015). Review on microbiota and effectiveness of probiotics use in older. *World J. Clin. Cases* 3 156–162. 10.12998/wjcc.v3.i2.156 25685762 PMC4317609

[B50] Saini A. Faulkner S. Al-Shanti N. Stewart C. (2009). Powerful signals for weak muscles. *Ageing Res. Rev.* 8 251–267. 10.1016/j.arr.2009.02.001 19716529

[B51] Scher J. U. Sczesnak A. Longman R. S. Segata N. Ubeda C. Bielski C. (2013). Expansion of intestinal Prevotella copri correlates with enhanced susceptibility to arthritis. *Elife* 2:e01202. 10.7554/eLife.01202 PMC3816614 24192039

[B52] Sellami M. Gasmi M. Denham J. Hayes L. D. Stratton D. Padulo J. (2018). Effects of Acute and Chronic Exercise on Immunological Parameters in the Elderly Aged: can Physical Activity Counteract the Effects of Aging? *Front. Immunol.* 9:2187. 10.3389/fimmu.2018.02187 30364079 PMC6191490

[B53] Shankar V. Gouda M. Moncivaiz J. Gordon A. Reo N. V. Hussein L. (2017). Differences in Gut Metabolites and Microbial Composition and Functions between Egyptian and U.S. Children Are Consistent with Their Diets. *mSystems.* 2 e00169–16. 10.1128/mSystems.00169-16 PMC5296411 28191503

[B54] Shephard R. J. (2009). Maximal oxygen intake and independence in old age. *Br. J. Sports Med.* 43 342–346. 10.1136/bjsm.2007.044800 18403414

[B55] UK Chief Medical Officers (CMO). (2019). *UK Chief Medical Officers’ Physical Activity Guidelines.* Available online at: https://assets.publishing.service.gov.uk/government/uploads/system/uploads/attachment_data/file/832868/uk-chief-medical-officers-physical-activity-guidelines.pdf (accessed Dec 12, 2020).

[B56] Vaiserman A. M. Koliada A. K. Marotta F. (2017). Gut microbiota: a player in aging and a target for anti-aging intervention. *Ageing Res. Rev.* 35 36–45. 10.1016/j.arr.2017.01.001 28109835

[B57] Xu C. Zhu H. Qiu P. (2019). Aging progression of human gut microbiota. *BMC Microbiol.* 19:236. 10.1186/s12866-019-1616-2 31660868 PMC6819604

[B58] Yang Y. Shi Y. Wiklund P. Tan X. Wu N. Zhang X. (2017). The Association between Cardiorespiratory Fitness and Gut Microbiota Composition in Premenopausal Women. *Nutrients* 9:792. 10.3390/nu9080792 28757576 PMC5579588

[B59] Yin J. Liao S.-X. He Y. Wang S. Xia G.-H. Liu F.-T. (2015). Dysbiosis of Gut Microbiota With Reduced Trimethylamine-N-Oxide Level in Patients With Large-Artery Atherosclerotic Stroke or Transient Ischemic Attack. *J. Am. Heart Assoc.* 4:e002699. 10.1161/JAHA.115.002699 PMC4845212 26597155

[B60] Zhang L. Wu Y.-N. Chen T. Ren C.-H. Li X. Liu G.-X. (2019). Relationship between intestinal microbial dysbiosis and primary liver cancer. *Hepatobiliary Pancreat. Dis. Int.* 18 149–157. 10.1016/j.hbpd.2019.01.002 30661942

[B61] Zhong X. Harrington J. M. Millar S. R. Perry I. J. O’Toole P. W. Phillips C. M. (2020). Gut Microbiota Associations with Metabolic Health and Obesity Status in Older Adults. *Nutrients* 12:2364. 10.3390/nu12082364 32784721 PMC7468966

