# Supplementary figures and images for: Strenuous Physical Training, Physical Fitness, Body Composition and Bacteroides to Prevotella Ratio in the Gut of Elderly Athletes
Source: Front Physiol. 2021 Jun 22;12:670989. doi: 10.3389/fphys.2021.670989 (PMC8257935; doi:10.3389/fphys.2021.670989)

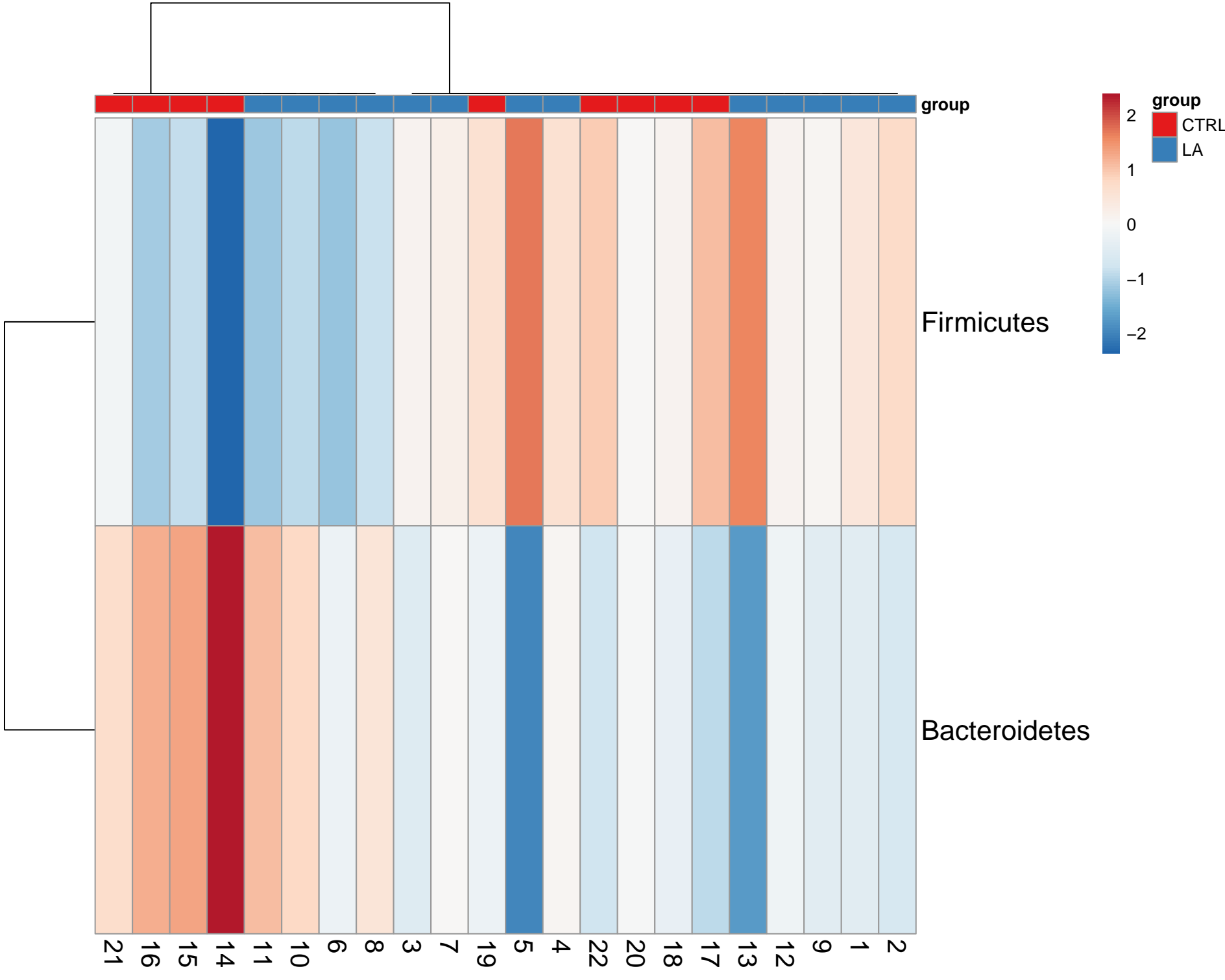

Supplement: Supplementary file 1 [file Image_1.pdf]

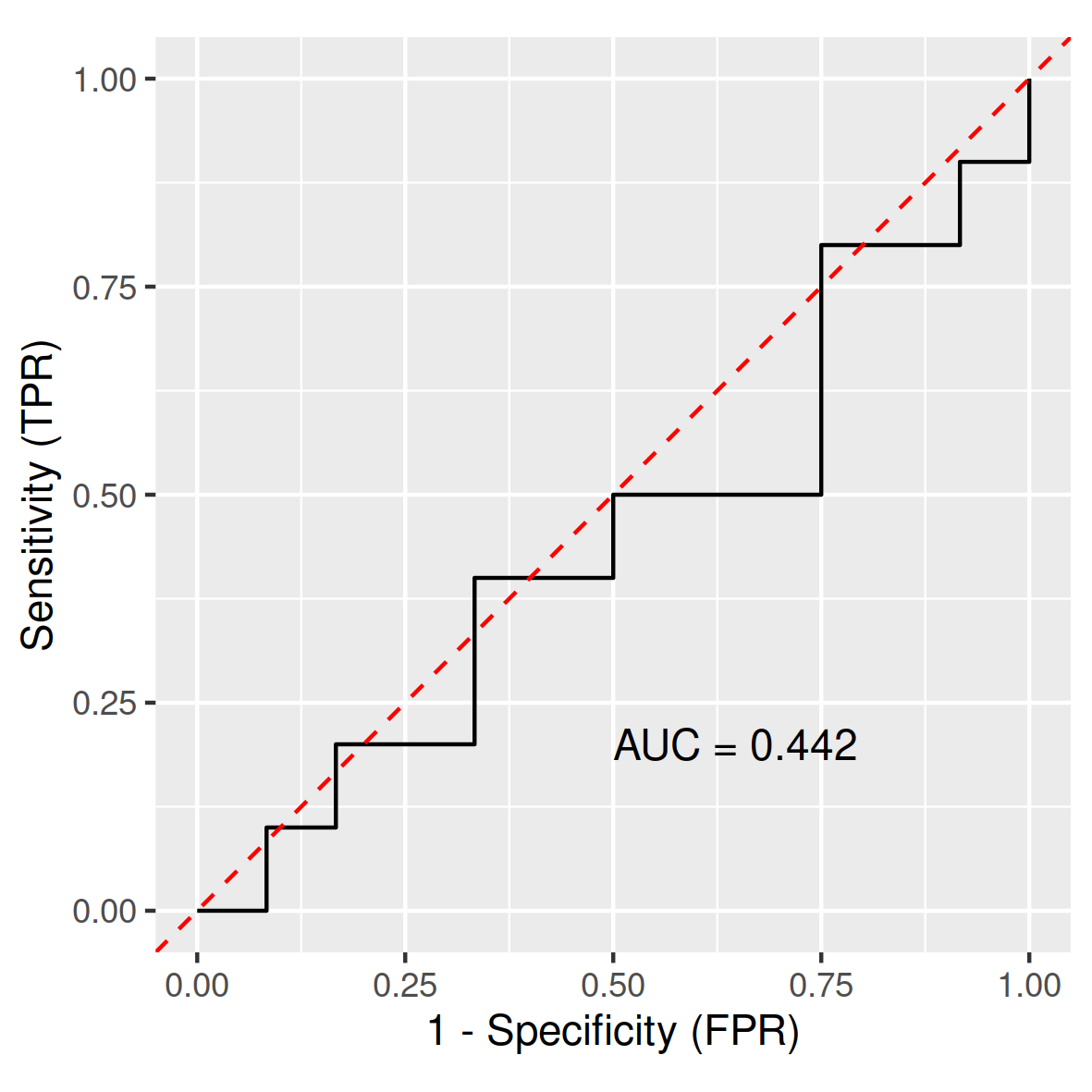

Supplement: Supplementary file 2 [file Image_2.tiff]
